# Biogeochemical and Microbial Variation across 5500 km of Antarctic Surface Sediment Implicates Organic Matter as a Driver of Benthic Community Structure

**DOI:** 10.3389/fmicb.2016.00284

**Published:** 2016-03-23

**Authors:** Deric R. Learman, Michael W. Henson, J. Cameron Thrash, Ben Temperton, Pamela M. Brannock, Scott R. Santos, Andrew R. Mahon, Kenneth M. Halanych

**Affiliations:** ^1^Department of Biology, Institute for Great Lakes Research, Central Michigan UniversityMt. Pleasant, MI, USA; ^2^Department of Biological Sciences, Louisiana State UniversityBaton Rouge, LA, USA; ^3^Department of Biosciences, University of ExeterExeter, UK; ^4^Department of Biological Sciences, Auburn UniversityAuburn, AL, USA

**Keywords:** benthic communities, Antarctica, aquatic microbiology, biogeochemistry, microbial ecology

## Abstract

Western Antarctica, one of the fastest warming locations on Earth, is a unique environment that is underexplored with regards to biodiversity. Although pelagic microbial communities in the Southern Ocean and coastal Antarctic waters have been well-studied, there are fewer investigations of benthic communities and most have a focused geographic range. We sampled surface sediment from 24 sites across a 5500 km region of Western Antarctica (covering the Ross Sea to the Weddell Sea) to examine relationships between microbial communities and sediment geochemistry. Sequencing of the 16S and 18S rRNA genes showed microbial communities in sediments from the Antarctic Peninsula (AP) and Western Antarctica (WA), including the Ross, Amundsen, and Bellingshausen Seas, could be distinguished by correlations with organic matter concentrations and stable isotope fractionation (total organic carbon; TOC, total nitrogen; TN, and δ^13^C). Overall, samples from the AP were higher in nutrient content (TOC, TN, and NH_4_^+^) and communities in these samples had higher relative abundances of operational taxonomic units (OTUs) classified as the diatom, *Chaetoceros*, a marine cercozoan, and four OTUs classified as *Flammeovirgaceae* or *Flavobacteria*. As these OTUs were strongly correlated with TOC, the data suggests the diatoms could be a source of organic matter and the *Bacteroidetes* and cercozoan are grazers that consume the organic matter. Additionally, samples from WA have lower nutrients and were dominated by *Thaumarchaeota*, which could be related to their known ability to thrive as lithotrophs. This study documents the largest analysis of benthic microbial communities to date in the Southern Ocean, representing almost half the continental shoreline of Antarctica, and documents trophic interactions and coupling of pelagic and benthic communities. Our results indicate potential modifications in carbon sequestration processes related to change in community composition, identifying a prospective mechanism that links climate change to carbon availability.

## Introduction

Changing climate in Antarctica has potential to initiate a domino effect that could impact ecosystems from continental ice sheets to the seafloor. Recent research has shown the West Antarctic Ice Sheet (WAIS) is one of the fastest warming locations on Earth (Bromwich et al., 2013; Hillenbrand et al., 2013). Consequently, the WAIS has been losing ice mass (Rignot et al., 2011; Pritchard et al., 2012; Shepherd et al., 2012; Depoorter et al., 2013), which not only has the potential to cause changes in sea-level (Bindschadler, 2006), but also to influence water temperature, salinity (Arneborg et al., 2012 and references therein) and nutrient cycling. The melt season is known to stimulate primary productivity (Smith and Gordon, 1997; Arrigo et al., 1998; Ducklow et al., 2006; Smith et al., 2007), which then increases the amount of organic matter sourced from the water column to sediments (Billett et al., 1983; Wefer et al., 1988; Honjo et al., 2000; Kennedy et al., 2002; Ducklow et al., 2006; Gillies et al., 2012). Further, increasing melt from the continental ice sheet would introduce more terrestrial carbon into marine and benthic environments, altering the quantity and type of carbon. Thus, a greater understanding of Antarctic ecosystems is essential to predict how changing climate will influence organic fluxes between benthic and pelagic communities.

Microbial marine communities play an important role in mediating nutrient cycling to both the water column (reviewed in Arrigo, 2005) and the deep biosphere (reviewed in Edwards et al., 2012) and diversity in these sediments is sensitive to environmental changes. Microbial sediment communities are important as they have been shown to play a major role in carbon cycling (Mayor et al., 2012), in addition to other biogeochemical cycles, such as sulfur, nitrogen, and phosphorus (Azam and Malfatti, 2007; Jorgensen and Boetius, 2007; Falkowski et al., 2008; Edwards et al., 2012). Further, marine sediment communities can be impacted by various chemical and physical parameters (Austen et al., 2002; Schauer et al., 2010; Zinger et al., 2011; Bienhold et al., 2012; Durbin and Teske, 2012; Liu et al., 2015; Nguyen and Landfald, 2015). In Antarctica, recent studies of planktonic marine microbial communities have shown beta diversity is correlated to both physical oceanographic (Wilkins et al., 2013) and chemical variation (Luria et al., 2014; Signori et al., 2014). Sediments from the Ross Sea (Carr et al., 2013) and the Drake Passage at the Antarctic Polar Front (Ruff et al., 2014) have shown organic matter can increase estimates of microbial abundance based on phospholipids and DNA sequencing, respectively. Another study examining sediments from the Southern Ocean (Jamieson et al., 2013) has shown organic matter did not impact species richness. Since climate change in Antarctica will alter the flow of organic matter and nutrients to benthic sediments, understanding how these changes will impact diversity is essential to predicting change in ecosystem functions.

In the present study, we examine the relationships between organic matter, nutrient content, and sediment microbial diversity. While several studies have examined microbial diversity in Antarctic sediments (e.g., Bowman and McCuaig, 2003; Bowman et al., 2003; Powell et al., 2003; Baldi et al., 2010; Carr et al., 2013, 2015; Jamieson et al., 2013; Ruff et al., 2014), each has used various sequencing methods, collected different types of geochemical parameters, and some have had a focused geographic scope. In contrast, we collected 24 sediment samples over a 5500 km transect of Western Antarctica that spans the Ross to the Weddell Seas. Small subunit (SSU) rRNA genes from all three domains of life were sequenced via Illumina MiSeq and correlated with geochemical and nutrient data. The total dataset greatly expands our existing understanding of benthic Antarctic sediments communities, and demonstrates important correlations between organic matter and sediment microbial diversity.

## Materials and experimental methods

### Sampling details

**Figure 1:**
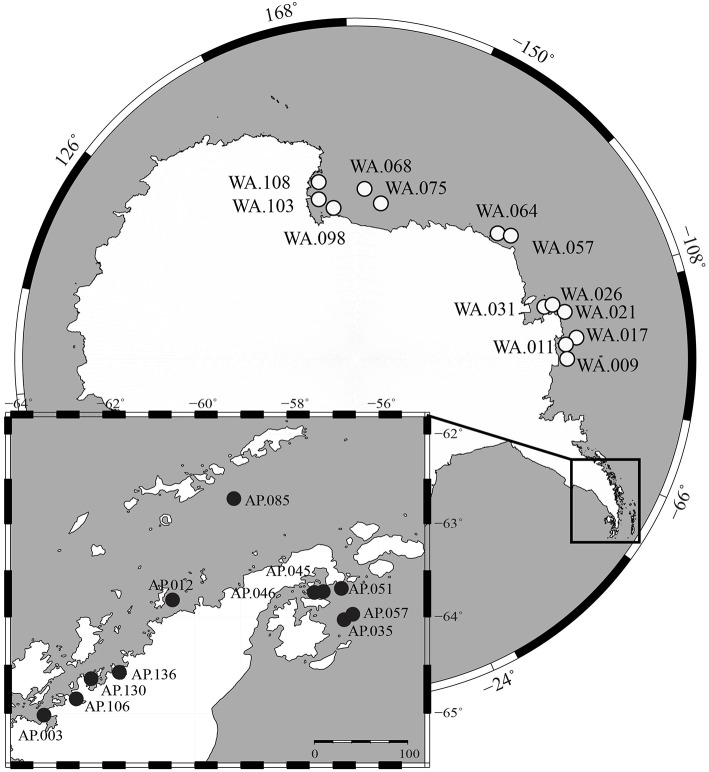
**Map of sample locations from Western Antarctica (WA, white circles) and Antarctic Peninsula (AP, black circles)**.

Surface sediment samples were collected from the continental shelf of Antarctica during two research cruises. The first cruise (Dec. 2013–Feb. 2014, *RVIB Nathaniel B. Palmer*) sampled Western Antarctica (WA), which includes the Amundsen Sea, Bellingshausen Sea, and Ross Sea, using a multicorer (Figure 1). The second cruise (Nov.–Dec. 2014, *ASRV Laurence M. Gould*) sampled the Antarctic Peninsula (AP) using a box corer. Samples were collected on the Antarctic shelf at depths ranging from 223 to 820 m. The top 3 cm of sediments were transferred into sampling tubes and stored frozen (−80°C). Samples were shipped frozen to Central Michigan University (CMU) within 3 months of collection. More details about sampling locations are found in Table S1.

### Sediment chemical analysis

Sediment samples were homogenized and shipped to EcoCore Analytical Services at Colorado State University for stable isotope (^13^C and ^15^N) and percent nitrogen and organic carbon analyses. Macronutrient (total organic carbon; TOC, total nitrogen; TN, NO_3_^−^, NH_4_^+^, S, P) analyses were conducted along with collection of available trace nutrient (e.g., metals) data at the Soil, Water, and Plant Testing Laboratory at Colorado State University (Table S1). The available fraction was defined as the metals that were extracted via Mehlich 3 acid digestions (Mehlich, 1984), and thus does not include an insoluble mineral component.

Correlations between chemical parameters were statistically examined via a Spearman's rank-order correlation in SPSS Statistics 22 (IBM, 2013). If a parameter had high correlation (*R* > 0.9 and significant at 0.01 level, two-tailed *t*-test) with another variable (%N and %TOC), then one parameter was removed from the dataset for downstream analysis (%N was removed). Also, data that were reported in percent or a ratio were transformed using an arcsine square root. Resulting nutrient data were then examined for broad trends via principal components analysis (PCA) in the statistical software PAST (Hammer et al., 2001).

### Microbial taxonomic analysis

DNA was extracted using a PowerSoil DNA extraction kit (MoBio) following the manufacturer's protocol. Approximately 4–8 extractions were completed on each sediment sample and pooled and concentrated with a DNA Clean and Concentrator kit (Zymo) due to low yields from some of the samples. DNA was then quantified using the Qubit2.0 Fluorometer (Life Technologies) and stored at −20°C. Bacterial and archaeal sequences were generated from the V4 region of 16S rRNA gene using the primer set 515f and 806r (Caporaso et al., 2012) while eukaryotic sequences were obtained with the V4 region of the 18S rRNA gene using the primer set 1391r and EukBR (Amaral-Zettler et al., 2009). Resulting amplicons were sequenced on an Illumina MiSeq as paired-end (PE) reads of 250-bp at Michigan State University's (MSU) Research Technology Support Facility (RTSF) Genomics Core. 16S and 18S rRNA gene amplicons were analyzed with Mothur v.1.33.3 (Kozich et al., 2013) using the Silva v119 database (Quast et al., 2013). Briefly, SSU 16S rRNA gene sequences were assembled into contigs and discarded if the contig had any ambiguous base pairs, possessed repeats greater than 8 bp, or were greater than 253 bp in length. Contigs were aligned using the Silva rRNA v119 database, checked for chimeras using UCHIME (Edgar et al., 2011), and classified using the Greengenes rRNA May 2013 database (DeSantis et al., 2003, 2006). Contigs classifying to chloroplast, eukaryotes, mitochondria, or “unknown” affinities were removed from the data and the remaining contigs were clustered into operational taxonomic units (OTUs) using a 0.03 dissimilarity threshold (OTU_0.03_).

Due to sequencing error, 18S rRNA reverse sequences were shorter than expected, causing little to no overlap of the paired end contigs. Therefore, only forward read sequences were used in downstream analyses (mean sequence length = 250 bp). Those classified as “unknown,” Archaea, or Bacteria were removed from the data and remaining contigs were clustered into OTUs using a 0.03 dissimilarity threshold (OTU_0.03_).

Data analyses of OTUs was done using the R statistical environment v3.2.1 (R Development Core Team, 2008), within the package PhyloSeq (McMurdie and Holmes, 2013). For estimating alpha-diversity, the filtered OTUs were used to calculate species richness using the “estimate_richness” command within PhyloSeq, which plots Simpson, Chao1, and Shannon diversity (McMurdie and Holmes, 2013, and references therein). After alpha diversity calculations were completed, potentially erroneous rare OTUs, those without at least a total of two sequences in two or more samples, were discarded. The amplicon reads were normalized using the package DESeq2 (Love et al., 2014) following the general procedure for normalization using a variance stabilizing transformation (see Supplemental Materials for all R code used). DESeq2 normalized reads were used for all downstream analyses. For 16S rRNA sequences, beta-diversity between samples was examined using Bray-Curtis distances and ordinated using non-metric multidimensional scaling (NMDS). For 18S rRNA sequences, the relative abundance counts were converted to a presence/absence matrix (due to the potential for eukaryotic organisms to be pluricellular) and beta diversity was calculated by generating Jaccard indices. Analysis of similarity (ANOSIM) was used to test the significance of differences between groups of samples (e.g., WP vs. AP) of the NMDS analyses. Correlation between measured geochemical and macronutrient (defined as TOC, TN, NO_3_^−^, NH_4_^+^, S, Fe, P) data and the beta-diversity data was investigated in R with the envfit function (Oksanen et al., 2013). In addition, environmental variables (pH, TOC, TN, NO_3_^−^, NH_4_^+^, S, P, Fe, Si, δ^15^N, and δ^13^C_org_) were tested for significance when compared to the axes of NDMS plots compared to beta-diversity variables to examine patterns between geochemical data and beta-diversity plots. Further, correlations between geochemical parameters and relative abundance of OTUs were statistically evaluated with Spearman's rank-order correlation (SPSS).

Raw rRNA reads (16S and 18S) have been submitted to the European Nucleotide Archive (study accession number: PRJEB11496 and PRJEB11497, respectively). A table with OTU relative abundances can be found in the Supplementary Materials.

## Results and discussion

### Relating organic matter (OM) and nutrients to benthic diversity

This study shows community structure in benthic sediments is correlated to nutrient content and also suggests a possible coupling between pelagic and benthic communities. We collected 24 benthic samples spanning a 5500 km region of Antarctica that includes Western Antarctica (WA) and the Antarctic Peninsula (AP; Figure 1). Sediment macronutrients (TOC, TN, NO_3_^−^, NH_4_^+^, S, Fe, P) were differentiated based on geographic region, with the general trend that samples from AP had relatively higher TOC, TN, and NH_4_^+^ compared to WA (Figure S1). Stable carbon and nitrogen isotopes were collected from sediments to provide insight into the provenance of the organic matter. The δ^13^C_org_-values found in the sediments ranged from −27.5 to −22.2%0 (average −24.6%0) and the δ^15^N-values range from 1.2 to 4.2%0 (combined average 3.0%0) (Figure S2). Other studies have shown phytoplankton and phytodetritus have δ^15^N-values from 3.2 to 7.9 and δ^13^C_org_-values from −14.94 to −33.93 (Meyers, 1994; Cloern et al., 2002; Mincks et al., 2008). As the ranges of these data is large, it is difficult to determine exact sources, however, the data collected here does suggest phytoplankton as a possible source of organic material to sediments. Samples from WA had, on average, significantly lower δ^13^C_org_-values than those from the AP (−25.5 and −23.4, respectively, *t*-test two-tailed *P* < 0.0001), suggesting WA samples had relatively more recalcitrant carbon. Overall, sediment geochemistry suggests organic matter predominantly sourced by phytoplankton deposition and degradation; however, samples from the AP had relatively higher quantities of macronutrients and more labile carbon. Since the AP is generally warmer than WA (Barnes et al., 2006), the possibility of a higher melting process could bring more nutrients into the water, which is one explanation for this variation. In addition, warmer temperatures could favor microbial activities and the remineralization of nutrients.

**Figure 2:**
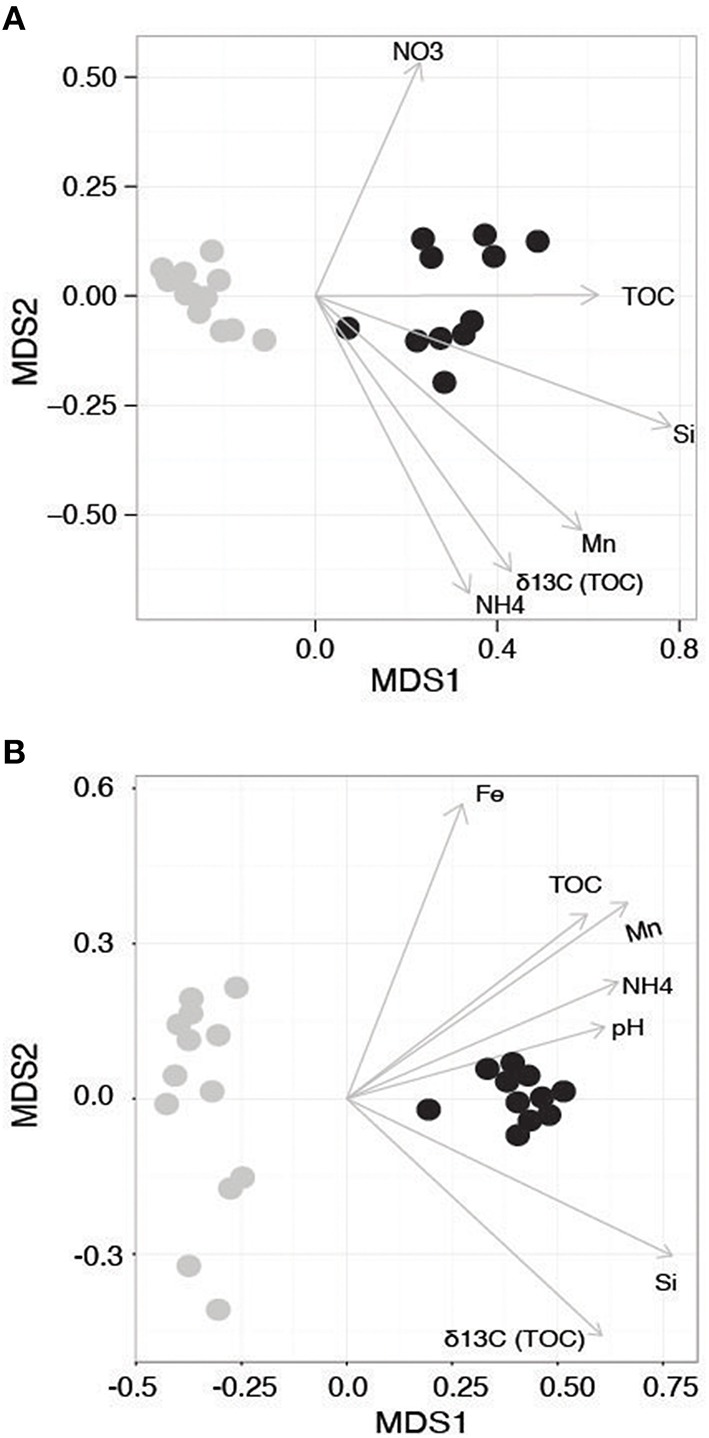
**Non-metric Multidimensional Scaling (NMDS) ordination of the beta diversity of the (A) 16S and (B) 18S rRNA-defined communities**. Samples from Western Antarctica (WA) are gray and Antarctic Peninsula (AP) samples are black. Arrows denote environmental gradients that statistically correlate with the ordination.

The 16S rRNA amplicon dataset included a total of 11,380 OTUs following filtering (initially 10,894,711 raw sequencing reads) and the 18S rRNA gene data generated a total of 4691 OTUs (initially 8,161,768 raw reads). Both 16S and 18S rRNA gene beta diversity ordinations showed communities strongly segregated according to the WA and AP collection regions and sampling time (Figure 2A, ANOSIM *R* = 0.993, *P* = 0.001 and Figure 2B ANOSIM *R* = 0.989, *P* = 0.001), driven by differences in macronutrients and organic matter (Figures 2A,B). The partitioning of diversity based on geographic region is also similar to the variation seen with the macronutrient data. Jamieson et al. (2013) examined the impact of chlorophyll content on Antarctica sediment communities and found few differences in bacterial diversity between sites with different chlorophyll content. However, the %N and TOC were similar between sampling sites, leading the authors to hypothesize that the minor variations found between them may be related to organic matter quality in addition to quantity. Quality of carbon might be a stronger driver in the present study as the samples from AP have relatively more liable carbon.

Prokaryotic communities from WA had on average >1.4x higher richness (Chao1 standard error range 13,075.6–26, 868.7) than those from the AP (Chao1.se range 9051.0–25,498.8, Figure S3A), potentially due to a large number of low abundance (less than two reads) OTUs in the WA samples. Previous 16S rRNA gene studies have calculated Chao1-values between 360 and 899 in the Southern Ocean (Jamieson et al., 2013), 1166 in surface sediment from the Ross Sea (Carr et al., 2013), and 2217 (OTUs calculated at 98% identity) in the Polar Front region (Ruff et al., 2014). Though some variation in richness estimations is seen, this can be attributed to the advent of improved next generation sequencing technologies (i.e., Illumina MiSeq) that has greatly increased the number of sequences garnered from amplicon studies and therefore increased the number of observed OTUs.

The driving force behind diversity and nutrient differences found in WA and AP is likely to be related to multiple, intertwined parameters. Taken together, geochemical and microbial diversity data here suggests a combination of the state (e.g., liable vs. recalcitrant) of organic matter and relative nutrient concentration influences Antarctic sediment communities. One possible explanation for the differences observed between WA and AP is that they were collected in two different austral summers. Both regions were sampled in the Astral Summer, during which particulate organic matter deposition begins and increases in the following months (Smith et al., 2008, 2012). However, organic matter deposition in Western Antarctica and the Antarctic Peninsula can be highly variable (Ducklow et al., 2006; Smith et al., 2006, 2008, 2012; Fragoso and Smith, 2012) and related to numerous factors such as temperature, sedimentation, ice cover, currents, and phytoplankton blooms (Arrigo et al., 1998; Ducklow et al., 2006; Smith et al., 2006).

### Shared community members across a 5500 km portion of Western Antarctica

**Figure 3:**
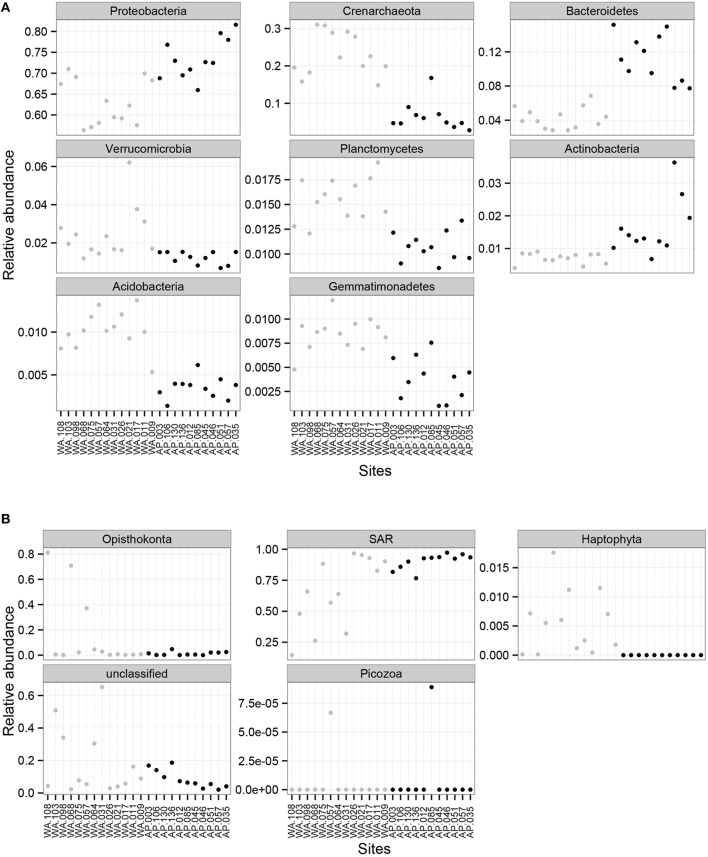
**Mean relative abundance of (A) 16S rRNA sequences based bacterial and archaeal phylum and (B) 18S rRNA sequences based on eukaryotic orders for Western Antarctica (WA, gray circles) and Antarctic Peninsula (AP, black circles)**.

**Figure 4:**
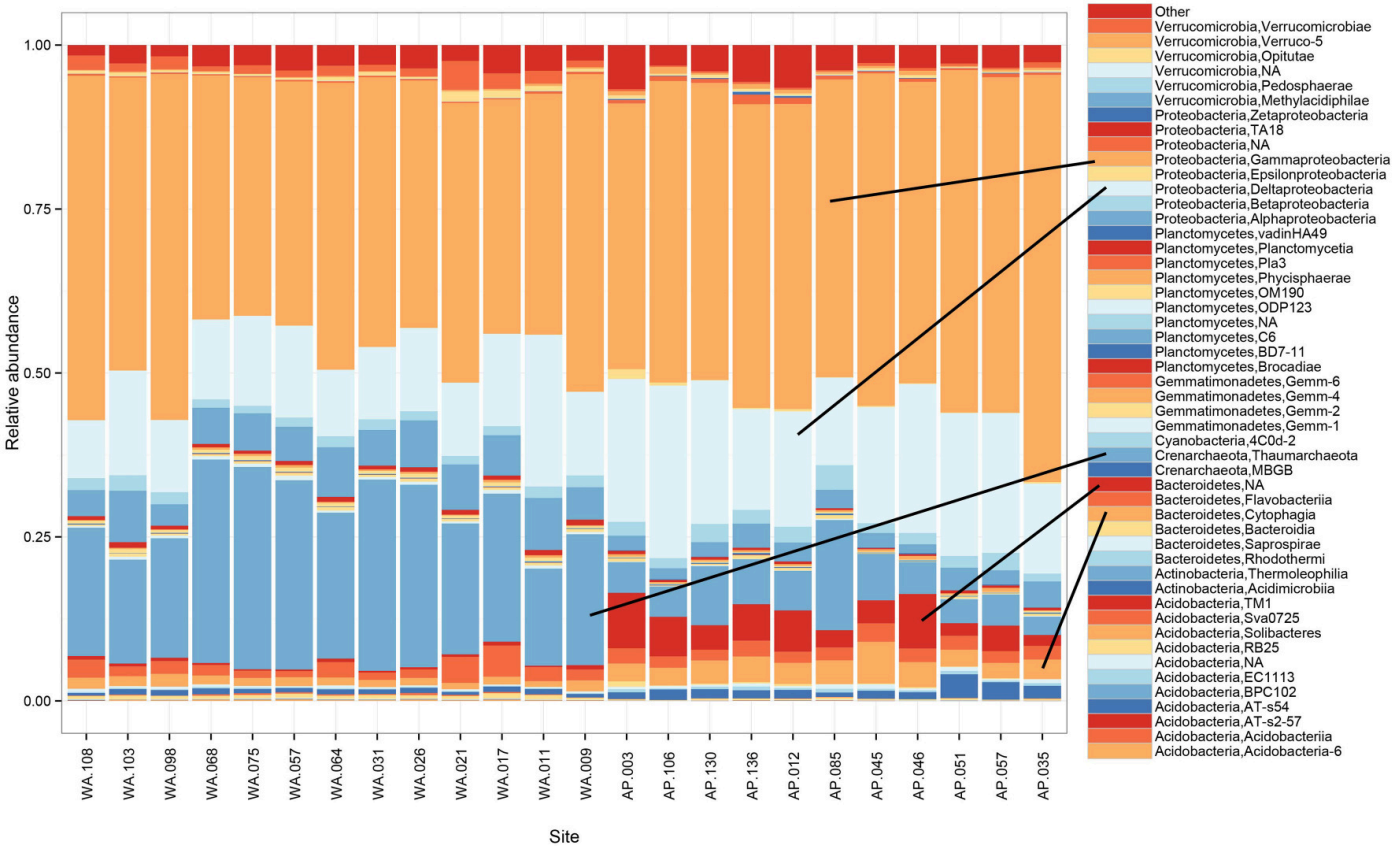
**Taxonomic composition and relative abundance of 16S rRNA sequences based on the class breakdown of the bacterial and archaea phyla that appeared at ≥1% relative abundance in any of the samples**.

*Proteobacteria* dominated the 16S rRNA sequenced communities of all sites, with major contributions (~15.5% of the sequenced community) from *Crenarchaeota*, primarily *Nitrosopumulis*-type Thaumarchaeota (these are classified as Phylum *Crenarchaeota* by the Greengenes database). Other OTUs were represented by organisms from the phyla *Bacteroidetes, Verrucomicrobia, Planctomycetes, Actinobacteria, Acidobacteria*, and *Gemmatimonadetes* (Figures 3A, 4), and minor contributions (phyla with < 0.52% relative abundance) from dozens of others (Figure S4). Relative abundances of *Thaumarchaeota, Planctomycetes, Acidobacteria*, and *Gemmatimonadetes* decreases along the WA to AP transit, whereas *Bacteriodetes* and *Actinobacteria* showed the opposite trend (Figure 3A, statistical differences calculated with a *t*-test, two-tailed *P* < 0.0001 for all mentioned other than *Actinobacteria*, *P* = 0.0010). Notable class level distinctions showed a relative abundance of *Gamma*- and *Delta-proteobacteria* across all samples, and an increase in *Cytophagia* of *Bacteriodetes* in the AP (Figure 4). Overall, the relative abundant phyla identified in these sediments have been seen in other studies on Antarctic sediments. Specifically, Ruff et al. (2014) documented sediment that were relatively abundant with *Proteobacteria* and various other studies (Bowman and McCuaig, 2003; Powell et al., 2003; Baldi et al., 2010) all found *Gamma*- and *Delta-proteobacteria* to be relatively dominant community members. In addition, the taxa documented in this study have also been identified as relatively abundant in marine sediments in general (Zinger et al., 2011). Notably, the 16S primer set used here has recently been shown to overestimate *Gammaproteobacteria* and under represent *Alphaproteobacteria* (see Parada et al., 2015), which could impact these data.

In general, the sequenced 16S rRNA communities of all benthic sediments had similar dominant OTUs. Both WA and AP shared three of the top five most abundant OTUs. OTU0000001, classified to the family of *Piscirickettsiaceae*, was the most dominate OTU in samples from AP (Figure S5A) and its relative abundance negatively correlated with δ^13^C_org_, NH_4_^+^, and Si (Table S2). Bacteria within the family *Piscirickettsiaceae* have been identified in planktonic communities (Giovannelli et al., 2012; Wang et al., 2015) and *Cycloclasticus pugetii* (found within the family *Piscirickettsiaceae*) has been shown to degrade complex carbon (Geiselbrecht et al., 1998; Kasai et al., 2002; Wang et al., 2008). OTU0000002, classified to the genus *Nitrosopumilus*, was extremely abundant (Figure S5A) and its relative abundance was statistically correlated with δ^13^C_org_ and Si (Table S2). All known isolates of *Nitrosopumilus* are lithotrophic archaea (Konneke et al., 2005, 2014; Walker et al., 2010; Qin et al., 2014), and these have been documented as dominant community members in Antarctic sediments in the Weddell Sea (Gillan and Danis, 2007), as well as in a variety of pelagic environments (Konneke et al., 2005; Labrenz et al., 2010; Baker et al., 2012). OTU0000004 (Figure S5A) is a member of the OM60/NOR5 clade and its relative abundance correlated with pH, δ^13^C_org_, NH_4_^+^, and Si (Table S2). OM60/NOR5 clade members are known inhabitants of surface water and coastal sediments (Connon and Giovannoni, 2002; Yan et al., 2009; Spring et al., 2013; Sharma et al., 2014). A recent study has also implicated members of this clade as key in degrading phytoplankton-derived organic matter in sediments of the Drake Passage at the Antarctic Polar Front (Ruff et al., 2014). The correlation of the *Piscirickettsiaceae* and OM60/NOR5 clade OTU to carbon isotope values and carbon quality might be related to the ability of each organism to metabolize carbon.

Overwhelmingly, the dominant organisms were members of the SAR (Stramenopiles, Alveolates, Rhizaria) supergroup (Burki et al., 2007), with some exceptions in WA (Figure 3B). Members of SAR have been found in marine environments, including the Antarctic water column (Luria et al., 2014) and sediments (Habura et al., 2004; Pawlowski et al., 2011). Other notable groups documented in the sediments were Opisthokonts and Haptophytes. Alpha diversity calculations show richness (Chao1) and evenness (Shannon and Simpson) values as being higher in WA then AP (Figure S3B).

### Nutrient quantity and quality drive relative abundance of dominant taxa

In general, the relative abundance of *Thaumarchaeota* was higher in samples from WA (Figure 4), which contained fewer organics and nutrients when compared to samples from AP (Figure S1). In addition, relative abundance of the class *Thaumarchaeota* and an abundant *Thaumarchaeota* OTU (OTU000003, most abundant OTU in WA) were both inversely correlated (0.01 level, two tailed *t*-test) with pH, δ^13^C_org_, NH_4_^+^, and Si (Table S2). Representatives from *Thaumarchaeota* have been previously reported in Antarctic marine environments (Delong et al., 1994; Murray et al., 1998; Alonso-Saez et al., 2012; Signori et al., 2014; Hernandez et al., 2015), sediments (Gillan and Danis, 2007), and soils (Ayton et al., 2010; Richter et al., 2014). Known *Thaumarchaeota* are lithotrophs capable of ammonium oxidation and some are known to degrade proteins (Leininger et al., 2006; Wuchter et al., 2006; Alonso-Saez et al., 2012; Luo et al., 2014). The overall low nutrient content in the sediments from WA and the statistical relationship between *Thaumarchaeota*, low TOC, and slightly more refractory carbon (e.g., lower δ^13^C_org_) suggests these are conditions that allow these organisms to flourish.

The relative abundance of the phylum *Bacteroidetes* correlated with TOC, Si, NH_4_^+^, and δ13C_org_ (Table S2). Of the five most abundant OTUs classifieds as *Bacteroidetes, four* were positively correlated with TOC, Si, and δ13C_org_ (Table S2) and the other was negatively correlated with pH, TOC, Si, and δ13C_org_ (Table S2). Bacteria from the *Bacteroidetes* phylum are very diverse and have been found in numerous environments from freshwater, soils, and oceans, and are expected to play a role in degrading complex carbon (reviewed in Kirchman, 2002; Gupta, 2004; Thomas et al., 2011).

Strikingly, the bulk of the measured SAR abundance across the AP could be accounted for by a single OTU (OTU0000013), designated SAR “unclassified” by GreenGenes taxonomy (Figure S5B). NCBI BLAST of the representative sequence for OTU0000013 returned hits with a 98% identity to the phylum *Cercozoan*. A marine cercozoan, *Cryothecomonas*, has been identified as a heterotroph that can feed on diatoms (Thomsen et al., 1991; Thaler and Lovejoy, 2012). Similarly, OTU0000010, designated as a *Stramenopile*, was only found in the AP. Examination of the representative OTU sequence for OTU000010 returned a 100% identity score to organisms classifying as *Chaetoceros*, the largest genus of diatoms. Conversely, an OTU (OTU0000030), classified as *Stramenopiles, Ochrophyta*, had the highest mean relative abundance in WA samples (Figure S5B), although its distribution was highly variable. In contrast to bacterial and archaeal data, relative abundance of broad phylogenetic eukaryotic categories was not significantly correlated with the nutrient data, however OTU0000013 (*Cercozoan*) and OTU0000010 (*Chaetoceros*), were correlated (0.01 level two tailed *t-test*) with pH, TOC, Si, and δ13C_org_ (Table S2).

Although, it is only speculation, the relationship between *Bacteroidetes* and SAR OTUs with TOC and Si could suggest a trophic relationship. Our data demonstrated an increased proportion of TOC, TN, NH_4_^+^, Si, and labile carbon in the AP relative to the WA samples, suggesting a recent diatom bloom and increased deposition of these components to the sea floor. Relative abundance of the phylum *Bacteroidetes* and four OTUs that fall under this phylum were also strongly correlated to TOC (Table S2). One of the OTUs that positively correlated with TOC was classified to the family *Flavobacteriaceae*. Members of this family have been linked to organic matter degradation in Antarctic sediments (Bowman and McCuaig, 2003; Baldi et al., 2010) and the Southern Ocean, with their relative abundance positively correlated with chlorophyll (Abell and Bowman, 2005) and algal biomass (Ruff et al., 2014), and genomic and metagenomic evidence for a role in processing organic matter from algae (Bauer et al., 2006; Fernandez-Gomez et al., 2013; Williams et al., 2013). Thus, our isotopic and SSU sequence results are consistent with the hypothesis that AP sediment organics were sourced by diatom sedimentation with subsequent degradation by *Bacteroidetes* and *Cercozoan* taxa. While this is only one possible explanation, sampling may have captured the remnants of a phytoplankton bloom and the subsequent trophic interaction.

## Conclusions

This study offers a unique look at a spatially diverse sample set covering 5500 km of Antarctic surface sediment. Analyses revealed a diverse benthic microbial community that was highly variable throughout the two cruises and geographic regions. Though the cruises were conducted during two different field seasons, one of the possible drivers of the highly variable communities could be quality and quantity of organic matter (TOC and δ^13^C). WA was characterized by relatively more recalcitrant carbon and had a larger influence of archaea, specifically *Thaumarchaeota*. Additionally, AP was characterized by relatively higher organics and had a large presence of sequences corresponding to diatoms (e.g., *Chaetoceros*) and taxa from the phyla *Bacteroidetes* and *Cercozoan*, which have been known to be associated with degradation of the corresponding organics from sinking particles and fecal pellets from blooms and their associated grazers. In addition, similarities were found throughout the entire sample set as three of the top five OTUs documented in the 16S rRNA sequenced communities were shared: OTU0000001 (*Piscirickettsiaceae*), OTU0000002 (*Nitrosopumilus*), and OTU0000004 (OM60/NOR5 clade).

Future variability in ice coverage, light, temperature, and food web structure could have a profound influence on the amount of organic carbon reaching the bottom, thus influencing the benthic community structure and their associated functions. With the continued warming of the WAIS, the amount of melt water entering coastal water around the ice shelf is predicted to increase, which impacts both coastal and open ocean water composition (Dierssen et al., 2002; Rignot et al., 2011; Pritchard et al., 2012; Shepherd et al., 2012; Depoorter et al., 2013). This increase in melt water could lead to the increase in phytoplankton blooms in these areas (Smith and Gordon, 1997; Arrigo et al., 1998; Ducklow et al., 2006; Smith et al., 2007) and, therefore, could increase organic matter transport to the sea floor. These increases of organic matter may ultimately influence communities that were once composed predominately of lithotrophic organisms, as observed in WA samples, to ones often associated with degradation of increasing organic matter, as observed in AP samples. Thus, changes to these communities in the form of their taxonomic members and resulting impacts on global nutrient cycling must continue to be studied.

## Author contributions

DL, MH, JT, BT, and PB analyzed data and prepared figures and tables. AM, PB, and KH collected samples. All authors contributed to writing the paper.

## Funding

Funds through NSF Antarctic Program: AM (CMU: Award Number 1043670), KH, and SS (AU Award Number: 1043745) and from Central Michigan University Faculty Research and Creative Endeavors (FRCE) Committee and College of Science and Technology.

### Conflict of interest statement

The authors declare that the research was conducted in the absence of any commercial or financial relationships that could be construed as a potential conflict of interest.

## References

[B1] Abell G. C. J. Bowman J. P. (2005). Ecological and biogeographic relationships of class Flavobacteria in the Southern Ocean. FEMS Microbiol. Ecol. 51, 265–277. 10.1016/j.femsec.2004.09.001 16329875

[B2] Alonso-Saez L. Waller A. S. Mende D. R. Bakker K. Farnelid H. Yager P. L.. (2012). Role for urea in nitrification by polar marine Archaea. Proc. Natl. Acad. Sci. U.S.A. 109, 17989–17994. 10.1073/pnas.1201914109 23027926 PMC3497816

[B3] Amaral-Zettler L. A. McCliment E. A. Ducklow H. W. Huse S. M. (2009). A method for studying protistan diversity using massively parallel sequencing of V9 hypervariable regions of small-subunit ribosomal RNA genes. PLoS ONE 4:e6372. 10.1371/annotation/50c43133-0df5-4b8b-8975-8cc37d4f2f26 19633714 PMC2711349

[B4] Arneborg L. Wahlin A. K. Bjork G. Liljebladh B. Orsi A. H. (2012). Persistent inflow of warm water onto the central Amundsen shelf. Nat. Geosci. 5, 876–880. 10.1038/ngeo1644

[B5] Arrigo K. R. (2005). Marine microorganisms and global nutrient cycles. Nature 437, 349–355. 10.1038/nature04159 16163345

[B6] Arrigo K. R. Worthen D. Schnell A. Lizotte M. P. (1998). Primary production in Southern Ocean waters. J. Geophys. Res. Oceans 103, 15587–15600. 10.1029/98JC00930

[B7] Austen M. C. Lambshead P. J. D. Hutchings P. A. Boucher G. Snelgrove P. V. R. Heip C. (2002). Biodiversity links above and below the marine sediment-water interface that may influence community stability. Biodivers. Conserv. 11, 113–136. 10.1023/A:1014098917535

[B8] Ayton J. Aislabie J. Barker G. M. Saul D. Turner S. (2010). Crenarchaeota affiliated with group 1.1b are prevalent in coastal mineral soils of the Ross Sea region of Antarctica. Environ. Microbiol. 12, 689–703. 10.1111/j.1462-2920.2009.02111.x 20002141

[B9] Azam F. Malfatti F. (2007). Microbial structuring of marine ecosystems. Nat. Rev. Microbiol. 5, 782–791. 10.1038/nrmicro1747 17853906

[B10] Baker B. J. Lesniewski R. A. Dick G. J. (2012). Genome-enabled transcriptomics reveals archaeal populations that drive nitrification in a deep-sea hydrothermal plume. Isme J. 6, 2269–2279. 10.1038/ismej.2012.64 22695863 PMC3504958

[B11] Baldi F. Marchetto D. Pini F. Fani R. Michaud L. Lo Giudice A. (2010). Biochemical and microbial features of shallow marine sediments along the Terra Nova Bay (Ross Sea, Antarctica). Cont. Shelf Res. 30, 1614–1625. 10.1016/j.csr.2010.06.009

[B12] Barnes D. K. A. Fuentes V. Clarke A. Schloss I. R. Wallace M. I. (2006). Spatial and temporal variation in shallow seawater temperatures around Antarctica. Deep Sea Res. I Top. Stud. Oceanogr. 53, 853–865. 10.1016/j.dsr2.2006.03.008

[B13] Bauer M. Kube M. Teeling H. Richter M. Lombardot T. Allers E. (2006). Whole genome analysis of the marine *Bacteroidetes* '*Gramella forsetii*' reveals adaptations to degradation of polymeric organic matter. Environ. Microbiol. 8, 2201–2213. 10.1111/j.1462-2920.2006.01152.x 17107561

[B14] Bienhold C. Boetius A. Ramette A. (2012). The energy-diversity relationship of complex bacterial communities in Arctic deep-sea sediments. Isme J. 6, 724–732. 10.1038/ismej.2011.140 22071347 PMC3309351

[B15] Billett D. Lampitt R. Rice A. Mantoura R. (1983). Seasonal sedimentation of phytoplankton to the deepsea benthos. Nature 302, 520–522. 10.1038/302520a0

[B16] Bindschadler R. (2006). The environment and evolution of the West Antarctic ice sheet: setting the stage. Phil. Trans. R. Soc. A 364, 1583–1605. 10.1098/rsta.2006.1790 16782601

[B17] Bowman J. P. McCammon S. A. Gibson J. A. Robertson L. Nichols P. D. (2003). Prokaryotic metabolic activity and community structure in Antarctic continental shelf sediments. Appl. Environ. Microbiol. 69, 2448–2462. 10.1128/AEM.69.5.2448-2462.2003 12732510 PMC154502

[B18] Bowman J. P. McCuaig R. D. (2003). Biodiversity, community structural shifts, and biogeography of prokaryotes within Antarctic continental shelf sediment. Appl. Environ. Microbiol. 69, 2463–2483. 10.1128/AEM.69.5.2463-2483.2003 12732511 PMC154503

[B19] Bromwich D. H. Nicolas J. P. Monaghan A. J. Lazzara M. A. Keller L. M. Weidner G. A. (2013). Central West Antarctica among the most rapidly warming regions on Earth. Nat. Geosci. 6, 139–145. 10.1038/ngeo1671

[B20] Burki F. Shalchian-Tabrizi K. Minge M. Skjaeveland A. Nikolaev S. I. Jakobsen K. S.. (2007). Phylogenomics reshuffles the eukaryotic supergroups. PLoS ONE 2:e790. 10.1371/journal.pone.0000790 17726520 PMC1949142

[B21] Caporaso J. G. Lauber C. L. Walters W. A. Berg-Lyons D. Huntley J. Fierer N.. (2012). Ultra-high-throughput microbial community analysis on the Illumina HiSeq and MiSeq platforms. Isme J. 6, 1621–1624. 10.1038/ismej.2012.8 22402401 PMC3400413

[B22] Carr S. A. Orcutt B. N. Mandernack K. W. Spear J. R. (2015). Abundant Atribacteria in deep marine sediment from the Adelie Basin, Antarctica. Front. Microbiol. 6:872. 10.3389/fmicb.2015.00872 26379647 PMC4549626

[B23] Carr S. A. Vogel S. W. Dunbar R. B. Brandes J. Spear J. R. Levy R.. (2013). Bacterial abundance and composition in marine sediments beneath the Ross Ice Shelf, Antarctica. Geobiology 11, 377–395. 10.1111/gbi.12042 23682649

[B24] Cloern J. E. Canuel E. A. Harris D. (2002). Stable carbon and nitrogen isotope composition of aquatic and terrestrial plants of the San Francisco Bay estuarine system. Limnol. Oceanogr. 47, 713–729. 10.4319/lo.2002.47.3.0713

[B25] Connon S. A. Giovannoni S. J. (2002). High-throughput methods for culturing microorganisms in very-low-nutrient media yield diverse new marine isolates. Appl. Environ. Microbiol. 68, 3878–3885. 10.1128/AEM.68.8.3878-3885.2002 12147485 PMC124033

[B26] Delong E. F. Wu K. Y. Prezelin B. B. Jovine R. V. M. (1994). High abundance of Archaea in Antarctic marine Picoplankton. Nature 371, 695–697. 10.1038/371695a0 7935813

[B27] Depoorter M. A. Bamber J. L. Griggs J. A. Lenaerts J. T. Ligtenberg S. R. van den Broeke M. R.. (2013). Calving fluxes and basal melt rates of Antarctic ice shelves. Nature 502, 89–92. 10.1038/nature12567 24037377

[B28] DeSantis T. Z. Dubosarskiy I. Murray S. R. Andersen G. L. (2003). Comprehensive aligned sequence construction for automated design of effective probes (CASCADE-P) using 16S rDNA. Bioinformatics 19, 1461–1468. 10.1093/bioinformatics/btg200 12912825

[B29] DeSantis T. Z. Hugenholtz P. Larsen N. Rojas M. Brodie E. L. Keller K.. (2006). Greengenes, a chimera-checked 16S rRNA gene database and workbench compatible with ARB. Appl. Environ. Microbiol. 72, 5069–5072. 10.1128/AEM.03006-05 16820507 PMC1489311

[B30] Dierssen H. M. Smith R. C. Vernet M. (2002). Glacial meltwater dynamics in coastal waters west of the Antarctic peninsula. Proc. Natl. Acad. Sci. U.S.A. 99, 1790–1795. 10.1073/pnas.032206999 11830636 PMC122272

[B31] Ducklow H. W. Fraser W. Karl D. M. Quetin L. B. Ross R. M. Smith R. C. (2006). Water-column processes in the West Antarctic Peninsula and the Ross Sea: interannual variations and foodweb structure. Deep Sea Res. II 53, 834–852. 10.1016/j.dsr2.2006.02.009

[B32] Durbin A. M. Teske A. (2012). Archaea in organic-lean and organic-rich marine subsurface sediments: an environmental gradient reflected in distinct phylogenetic lineages. Front. Microbiol. 3:168. 10.3389/fmicb.2012.00168 22666218 PMC3364523

[B33] Edgar R. C. Haas B. J. Clemente J. C. Quince C. Knight R. (2011). UCHIME improves sensitivity and speed of chimera detection. Bioinformatics 27, 2194–2200. 10.1093/bioinformatics/btr381 21700674 PMC3150044

[B34] Edwards K. J. Becker K. Colwell F. (2012). The deep, dark energy biosphere: intraterrestrial Life on Earth. Annu. Rev. Earth Planet. Sci. 40, 551–568. 10.1146/annurev-earth-042711-105500

[B35] Falkowski P. G. Fenchel T. Delong E. F. (2008). The microbial engines that drive Earth's biogeochemical cycles. Science 320, 1034–1039. 10.1126/science.1153213 18497287

[B36] Fernandez-Gomez B. Richter M. Schuler M. Pinhassi J. Acinas S. G. Gonzalez J. M.. (2013). Ecology of marine Bacteroidetes: a comparative genomics approach. Isme J. 7, 1026–1037. 10.1038/ismej.2012.169 23303374 PMC3635232

[B37] Fragoso G. M. Smith W. O. (2012). Influence of hydrography on phytoplankton distribution in the Amundsen and Ross Seas, Antarctica. J. Mar. Syst. 89, 19–29. 10.1016/j.jmarsys.2011.07.008

[B38] Geiselbrecht A. D. Hedlund B. P. Tichi M. A. Staley J. T. (1998). Isolation of marine polycyclic aromatic hydrocarbon (PAH)-degrading Cycloclasticus strains from the Gulf of Mexico and comparison of their PAH degradation ability with that of puget sound Cycloclasticus strains. Appl. Environ. Microbiol. 64, 4703–4710. 9835552 10.1128/aem.64.12.4703-4710.1998 PMC90912

[B39] Gillan D. C. Danis B. (2007). The archaebacterial communities in Antarctic bathypelagic sediments. Deep Sea Res. I. Top. Stud. Oceanogr. 54, 1682–1690. 10.1016/j.dsr2.2007.07.002

[B40] Gillies C. L. Stark J. S. Johnstone G. J. Smith S. D. A. (2012). Carbon flow and trophic structure of an Antarctic coastal benthic community as determined by d13C and d15N. Estuar. Coast. Shelf Sci. 97, 44–57. 10.1016/j.ecss.2011.11.003

[B41] Giovannelli D. Grosche A. Starovoytov V. Yakimov M. Manini E. Vetriani C. (2012). *Galenea microaerophila* gen. nov., sp nov., a mesophilic, microaerophilic, chemosynthetic, thiosulfate-oxidizing bacterium isolated from a shallow-water hydrothermal vent. Int. J. Syst. Evol. Microbiol. 62, 3060–3066. 10.1099/ijs.0.040808-0 22307509

[B42] Gupta R. S. (2004). The phylogeny and signature sequences characteristics of Fibrobacteres, Chlorobi, and Bacteroidetes. Crit. Rev. Microbiol. 30, 123–143. 10.1080/10408410490435133 15239383

[B43] Habura A. Pawlowski J. Hanes S. D. Bowser S. S. (2004). Unexpected foraminiferal diversity revealed by small-subunit rDNA analysis of Antarctic sediment. J. Eukaryot. Microbiol. 51, 173–179. 10.1111/j.1550-7408.2004.tb00542.x 15134252

[B44] Hammer Ø. Harper D. A. T. Ryan P. D. (2001). PAST: Paleontological statistics software package for education and data analysis. Palaeontol. Electron. 4, 9.

[B45] Hernandez E. Piquet A. M. T. Lopez J. L. Buma A. G. J. Mac Cormack W. P. (2015). Marine archaeal community structure from Potter Cove, Antarctica: high temporal and spatial dominance of the phylum Thaumarchaeota. Polar Biol. 38, 117–130. 10.1007/s00300-014-1569-8

[B46] Hillenbrand C.-D. Kuhn G. Smith J. A. Gohl K. Graham A. G. C. Larter R. D. (2013). Grounding-line retreat of the West Antarctic Ice Sheet from inner Pine Island Bay. Geology 41, 35–38. 10.1130/G33469.1

[B47] Honjo S. Francois R. Manganini S. Dymond J. Collier R. (2000). Particle fluxes to the interior of the Southern Ocean in the Western Pacific sector along 170W. Deep Sea Res. II Top. Stud. Oceanogr. 47, 3521–3548. 10.1016/S0967-0645(00)00077-1

[B48] IBM (2013). IBM SPSS Statistics for Windows, 22 Edn. Armonk, NY: IBM Corp.

[B49] Jamieson R. E. Heywood J. L. Rogers A. D. Billett D. S. M. Pearce D. A. (2013). Bacterial biodiversity in deep-sea sediments from two regions of contrasting surface water productivity near the Crozet Islands, Southern Ocean. Deep Sea Res. I Oceanogr. Res. Papers 75, 67–77. 10.1016/j.dsr.2012.12.012

[B50] Jorgensen B. B. Boetius A. (2007). Feast and famine - microbial life in the deep-sea bed. Nat. Rev. Microbiol. 5, 770–781. 10.1038/nrmicro1745 17828281

[B51] Kasai Y. Kishira H. Harayama S. (2002). Bacteria belonging to the genus cycloclasticus play a primary role in the degradation of aromatic hydrocarbons released in a marine environment. Appl. Environ. Microbiol. 68, 5625–5633. 10.1128/AEM.68.11.5625-5633.2002 12406758 PMC129893

[B52] Kennedy H. Thomas D. N. Kattner G. Haas C. Dieckmann G. S. (2002). Particulate organic matter in Antarctic summer sea ice: concentration and stable isotopic composition. Mar. Ecol. Prog. Ser. 238, 1–13. 10.3354/meps238001

[B53] Kirchman D. L. (2002). The ecology of Cytophaga-Flavobacteria in aquatic environments. FEMS Microbiol. Ecol. 39, 91–100. 10.1016/s0168-6496(01)00206-9 19709188

[B54] Konneke M. Bernhard A. E. de la Torre J. R. Walker C. B. Waterbury J. B. Stahl D. A. (2005). Isolation of an autotrophic ammonia-oxidizing marine archaeon. Nature 437, 543–546. 10.1038/nature03911 16177789

[B55] Konneke M. Schubert D. M. Brown P. C. Hugler M. Standfest S. Schwander T.. (2014). Ammonia-oxidizing archaea use the most energy-efficient aerobic pathway for CO2 fixation. Proc. Natl. Acad. Sci. U.S.A. 111, 8239–8244. 10.1073/pnas.1402028111 24843170 PMC4050595

[B56] Kozich J. J. Westcott S. L. Baxter N. T. Highlander S. K. Schlossa P. D. (2013). Development of a dual-index sequencing strategy and curation pipeline for analyzing amplicon sequence data on the MiSeq illumina sequencing platform. Appl. Environ. Microbiol. 79, 5112–5120. 10.1128/AEM.01043-13 23793624 PMC3753973

[B57] Labrenz M. Sintes E. Toetzke F. Zumsteg A. Herndl G. J. Seidler M.. (2010). Relevance of a crenarchaeotal subcluster related to Candidatus Nitrosopumilus maritimus to ammonia oxidation in the suboxic zone of the central Baltic Sea. ISME J. 4, 1496–1508. 10.1038/ismej.2010.78 20535219

[B58] Leininger S. Urich T. Schloter M. Schwark L. Qi J. Nicol G. W.. (2006). Archaea predominate among ammonia-oxidizing prokaryotes in soils. Nature 442, 806–809. 10.1038/nature04983 16915287

[B59] Liu J. W. Liu X. S. Wang M. Qiao Y. L. Zheng Y. F. Zhang X. H. (2015). Bacterial and Archaeal communities in sediments of the North Chinese Marginal Seas. Microb. Ecol. 70, 105–117. 10.1007/s00248-014-0553-8 25501892

[B60] Love M. I. Huber W. Anders S. (2014). Moderated estimation of fold change and dispersion for RNA-seq data with DESeq2. Genome Biol. 15, 550. 10.1186/s13059-014-0550-8 25516281 PMC4302049

[B61] Luo H. Tolar B. B. Swan B. K. Zhang C. L. Stepanauskas R. Ann Moran M.. (2014). Single-cell genomics shedding light on marine Thaumarchaeota diversification. ISME J. 8, 732–736. 10.1038/ismej.2013.202 24196320 PMC3930325

[B62] Luria C. M. Ducklow H. W. Amaral-Zettler L. A. (2014). Marine bacterial, archaeal and eukaryotic diversity and community structure on the continental shelf of the western Antarctic Peninsula. Aquat. Microb. Ecol. 73, 107–121. 10.3354/ame01703

[B63] Mayor D. J. Thornton B. Hay S. Zuur A. F. Nicol G. M. McWilliam J. M.. (2012). Resource quality affects carbon cycling in deep-sea sediments. ISME J. 6, 1740–1748. 10.1038/ismej.2012.14 22378534 PMC3498925

[B64] McMurdie P. J. Holmes S. (2013). phyloseq: an R package for reproducible interactive analysis and graphics of microbiome census data. PLoS ONE 8:e61217. 10.1371/journal.pone.0061217 23630581 PMC3632530

[B65] Mehlich A. (1984). Mehlich-3 soil test extractant - a modification of Mehlich-2 extractant. Commun. Soil Sci. Plant Anal. 15, 1409–1416. 10.1080/00103628409367568

[B66] Meyers P. A. (1994). Preservation of elemental and isotopic source identification of sedimentary organic matter. Chem. Geol. 114, 289–302. 10.1016/0009-2541(94)90059-0

[B67] Mincks S. L. Smith C. R. Jeffreys R. M. Sumida P. Y. G. (2008). Trophic structure on the West Antarctic Peninsula shelf: detritivory and benthic inertia revealed by d13C and d15N analysis. Deep Sea Res. II 55, 2502–2514. 10.1016/j.dsr2.2008.06.009

[B68] Murray A. E. Preston C. M. Massana R. Taylor L. T. Blakis A. Wu K.. (1998). Seasonal and spatial variability of bacterial and archaeal assemblages in the coastal waters near Anvers Island, Antarctica. Appl. Environ. Microbiol. 64, 2585–2595. 9647834 10.1128/aem.64.7.2585-2595.1998 PMC106430

[B69] Nguyen T. T. Landfald B. (2015). Polar front associated variation in prokaryotic community structure in Arctic shelf seafloor. Front. Microbiol. 6:17. 10.3389/fmicb.2015.00017 25667586 PMC4304239

[B70] Oksanen J. Blanchet F. G. Kindt R. Legendre P. Minchin P. R. O'Hara R. B. (2013). vegan: Community Ecology Package. R package version 2.0-8. Available online at: http://CRAN.R-project.org/package=vegan

[B71] Parada A. E. Needham D. M. Fuhrman J. A. (2015). Every base matters: assessing small subunit rRNA primers for marine microbiomes with mock communities, time series and global field samples. Environ. Microbiol. [Epub ahead of print]. 10.1111/1462-2920.13023 26271760

[B72] Pawlowski J. Fontaine D. da Silva A. A. Guiard J. (2011). Novel lineages of Southern Ocean deep-sea foraminifera revealed by environmental DNA sequencing. Deep Sea Res. I Top. Stud. Oceanogr. 58, 1996–2003. 10.1016/j.dsr2.2011.01.009

[B73] Powell S. M. Bowman J. P. Snape I. Stark J. S. (2003). Microbial community variation in pristine and polluted nearshore Antarctic sediments. FEMS Microbiol. Ecol. 45, 135–145. 10.1016/S0168-6496(03)00135-1 19719624

[B74] Pritchard H. D. Ligtenberg S. R. M. Fricker H. A. Vaughan D. G. van den Broeke M. R. Padman L. (2012). Antarctic ice-sheet loss driven by basal melting of ice shelves. Nature 484, 502–505. 10.1038/nature10968 22538614

[B75] Qin W. Amin S. A. Martens-Habbena W. Walker C. B. Urakawa H. Devol A. H.. (2014). Marine ammonia-oxidizing archaeal isolates display obligate mixotrophy and wide ecotypic variation. Proc. Natl. Acad. Sci. U.S.A. 111, 12504–12509. 10.1073/pnas.1324115111 25114236 PMC4151751

[B76] Quast C. Pruesse E. Yilmaz P. Gerken J. Schweer T. Yarza P.. (2013). The SILVA ribosomal RNA gene database project: improved data processing and web-based tools. Nucleic Acids Res. 41, D590–D596. 10.1093/nar/gks1219 23193283 PMC3531112

[B77] R Development Core Team (2008). R: A Language and Environment for Statistical Computing. R Foundation for Statistical Computing.

[B78] Richter I. Herbold C. W. Lee C. K. McDonald I. R. Barrett J. E. Cary S. C. (2014). Influence of soil properties on archaeal diversity and distribution in the McMurdo Dry Valleys, Antarctica. FEMS Microbiol. Ecol. 89, 347–359. 10.1111/1574-6941.12322 24646164

[B79] Rignot E. Mouginot J. Scheuchl B. (2011). Ice flow of the Antarctic ice sheet. Science 333, 1427–1430. 10.1126/science.1208336 21852457

[B80] Ruff S. E. Probandt D. Zinkann A. C. Iversen M. H. Klaas C. Wurzberg L. (2014). Indications for algae-degrading benthic microbial communities in deep-sea sediments along the Antarctic Polar Front. Deep Sea Res. I Top. Stud. Oceanogr. 108, 6–16. 10.1016/j.dsr2.2014.05.011

[B81] Schauer R. Bienhold C. Ramette A. Harder J. (2010). Bacterial diversity and biogeography in deep-sea surface sediments of the South Atlantic Ocean. ISME J. 4, 159–170. 10.1038/ismej.2009.106 19829317

[B82] Sharma A. K. Becker J. W. Ottesen E. A. Bryant J. A. Duhamel S. Karl D. M.. (2014). Distinct dissolved organic matter sources induce rapid transcriptional responses in coexisting populations of *Prochlorococcus, Pelagibacter* and the OM60 clade. Environ. Microbiol. 16, 2815–2830. 10.1111/1462-2920.12254 24118765

[B83] Shepherd A. Ivins E. R. Geruo A. Barletta V. R. Bentley M. J. Bettadpur S.. (2012). A reconciled estimate of ice-sheet mass balance. Science 338, 1183–1189. 10.1126/science.1228102 23197528

[B84] Signori C. N. Thomas F. Enrich-Prast A. Pollery R. C. Sievert S. M. (2014). Microbial diversity and community structure across environmental gradients in Bransfield Strait, Western Antarctic Peninsula. Front. Microbiol. 5:647. 10.3389/fmicb.2014.00647 25566198 PMC4267279

[B85] Smith C. R. DeMaster D. J. Thomas C. Srsen P. Grange L. Evrard V. (2012). Pelagic-benthic coupling, food banks, and climate change on the West Antarctic Peninsula Shelf. Oceanography 25, 188–201. 10.5670/oceanog.2012.94

[B86] Smith C. R. Mincks S. DeMaster D. J. (2006). A synthesis of bentho-pelagic coupling on the Antarctic shelf: food banks, ecosystem inertia and global climate change. Deep Sea Res. I Top. Stud. Oceanogr. 53, 875–894. 10.1016/j.dsr2.2006.02.001

[B87] Smith C. R. Mincks S. DeMaster D. J. (2008). The FOODBANCS project: introduction and sinking fluxes of organic carbon, chlorophyll-a and phytodetritus on the western Antarctic Peninsula continental shelf. Deep Sea Res. I Top. Stud. Oceanogr. 55, 2404–2414. 10.1016/j.dsr2.2008.06.001

[B88] Smith W. O. Jr. Gordon L. I. (1997). Hyperproductivity of the Ross Sea (Antarctica) polynya during austral spring. Geophys. Res. Lett. 24, 233–236. 10.1029/96GL03926

[B89] Smith K. L. Robison B. H. Helly J. J. Kaufmann R. S. Ruhl H. A. Shaw T. J.. (2007). Free-drifting icebergs: hot spots of chemical and biological enrichment in the Weddell Sea. Science 317, 478–482. 10.1126/science.1142834 17588896

[B90] Spring S. Riedel T. Sproer C. Yan S. Harder J. Fuchs B. M. (2013). Taxonomy and evolution of bacteriochlorophyll a-containing members of the OM60/NOR5 clade of marine gammaproteobacteria: description of *Luminiphilus syltensis* gen. nov., sp. nov., reclassification of *Haliea rubra* as *Pseudohaliea rubra* gen. nov., comb. nov., and emendation of *Chromatocurvus halotolerans*. BMC Microbiol. 13:118. 10.1186/1471-2180-13-118 23705883 PMC3679898

[B91] Thaler M. Lovejoy C. (2012). Distribution and diversity of a protist predator cryothecomonas (cercozoa) in Arctic marine waters. J. Eukaryot. Microbiol. 59, 291–299. 10.1111/j.1550-7408.2012.00631.x 22703332

[B92] Thomas F. Hehemann J. H. Rebuffet E. Czjzek M. Michel G. (2011). Environmental and gut Bacteroidetes: the food connection. Front. Microbiol. 2:93. 10.3389/fmicb.2011.00093 21747801 PMC3129010

[B93] Thomsen H. A. Buck K. R. Bolt P. A. Garrison D. L. (1991). Fine-structure and biology of *Cryothecomonas* Gen-Nov (*Protista incertae* sedis) from the ice biota. Canad. J. Zool. 69, 1048–1070. 10.1139/z91-150

[B94] Walker C. B. de la Torre J. R. Klotz M. G. Urakawa H. Pinel N. Arp D. J.. (2010). *Nitrosopumilus maritimus* genome reveals unique mechanisms for nitrification and autotrophy in globally distributed marine crenarchaea. Proc. Natl. Acad. Sci. U.S.A. 107, 8818–8823. 10.1073/pnas.0913533107 20421470 PMC2889351

[B95] Wang B. Lai Q. Cui Z. Tan T. Shao Z. (2008). A pyrene-degrading consortium from deep-sea sediment of the West Pacific and its key member *Cycloclasticus* sp. P1. Environ. Microbiol. 10, 1948–1963. 10.1111/j.1462-2920.2008.01611.x 18430013

[B96] Wang K. Zhang D. M. Xiong J. B. Chen X. X. Zheng J. L. Hu C. J.. (2015). Response of bacterioplankton communities to cadmium exposure in coastal water microcosms with high temporal variability. Appl. Environ. Microbiol. 81, 231–240. 10.1128/AEM.02562-14 25326310 PMC4272717

[B97] Wefer G. Fischer G. Futterer D. Gersonde R. (1988). Seasonal particle flux in the Bransfield Strait, Antarctica. Deep Sea Res. 35, 891–898. 10.1016/0198-0149(88)90066-0

[B98] Wilkins D. van Sebille E. Rintoul S. R. Lauro F. M. Cavicchioli R. (2013). Advection shapes Southern Ocean microbial assemblages independent of distance and environment effects. Nat. Commun. 4, 2457. 10.1038/ncomms3457 24036630

[B99] Williams T. J. Wilkins D. Long E. Evans F. DeMaere M. Z. Raftery M. J.. (2013). The role of planktonic flavobacteria in processing algal organic matter in coastal East Antarctica revealed using metagenomics and metaproteomics. Environ. Microbiol. 15, 1302–1317. 10.1111/1462-2920.12017 23126454

[B100] Wuchter C. Abbas B. Coolen M. J. Herfort L. van Bleijswijk J. Timmers P.. (2006). Archaeal nitrification in the ocean. Proc. Natl. Acad. Sci. U.S.A. 103, 12317–12322. 10.1073/pnas.0600756103 16894176 PMC1533803

[B101] Yan S. Fuchs B. M. Lenk S. Harder J. Wulf J. Jiao N. Z.. (2009). Biogeography and phylogeny of the NOR5/OM60 clade of *Gammaproteobacteria*. Syst. Appl. Microbiol. 32, 124–139. 10.1016/j.syapm.2008.12.001 19216045

[B102] Zinger L. Amaral-Zettler L. A. Fuhrman J. A. Horner-Devine M. C. Huse S. M. Welch D. B. M.. (2011). Global patterns of bacterial beta-diversity in seafloor and seawater ecosystems. PLoS ONE 6:e24570. 10.1371/journal.pone.0024570 21931760 PMC3169623

