# Supplementary material for: Biogeochemical and Microbial Variation across 5500 km of Antarctic Surface Sediment Implicates Organic Matter as a Driver of Benthic Community Structure
Source: Front Microbiol. 2016 Mar 23;7:284. doi: 10.3389/fmicb.2016.00284 (PMC4803750; doi:10.3389/fmicb.2016.00284)
Supplement: Supplementary file 1 [file Table1.PDF]

Supplementary Material

Table S1. Sample names, geographical data, and raw geochemical data

Site Names	Lat (S)	Lon (W)	Water Depth (m)	pH	Total Carbon (%)	Total Organic Carbon (%)	Total Inorganic Carbon (%)	Nitrogen (%)	δ13C (TC)	δ13C (TOC)	δ15N	C/N	NH_4-N (mg/kg)	NO_3-N (mg/kg)	Sulfur (mg/kg)
WA.009	-71.4234	-91.4268	412	7.4	0.25	0.13	0.11	0.02	-15.08	-24.88	DL	7.7	5.4	0.30	550
WA.011	-71.4197	-93.4818	672	7.4	0.96	0.43	0.54	0.05	-10.18	-24.27	2.60	9.9	4.9	0.42	551
WA.017	-70.4844	-95.1957	476	7.4	1.16	0.38	0.78	0.05	-4.24	-25.49	1.21	9.3	4.9	0.62	498
WA.021	-71.4505	-102.1594	471	7.3	1.49	0.35	1.15	0.04	-4.33	-26.30	DL	9.2	4.4	0.45	216
WA.026	-72.2974	-104.2981	597	7.3	0.57	0.52	0.05	0.09	-25.01	-25.74	3.43	6.8	4.4	0.32	573
WA.031	-72.4601	-104.3554	572	7.4	0.58	0.55	0.04	0.08	-25.30	-25.89	3.45	7.7	4.6	0.27	574
WA.057	-73.2957	-129.5568	510	7.2	0.66	0.63	0.03	0.10	-24.14	-24.78	4.06	7.5	6.7	0.30	307
WA.064	-73.1582	-129.2692	478	7.3	0.63	0.57	0.07	0.08	-22.00	-24.80	4.13	8.1	7.3	1.40	604
WA.068	-75.1981	-176.5927	567	7.5	0.47	0.32	0.15	0.04	-16.08	-26.39	DL	10.4	6.6	0.50	163
WA.075	-76.2003	-170.5103	531	7.3	0.47	0.44	0.04	0.06	-24.57	-26.18	1.97	8.2	6.8	0.34	443
WA.098	-76.5502	170.0004	765	7.3	0.70	0.69	0.00	0.09	-26.57	-27.49	2.13	8.9	7.3	0.45	187
WA.103	-75.4987	166.2995	552	7.3	0.94	0.85	0.08	0.13	-24.11	-24.41	3.02	7.6	5.6	0.30	490
WA.108	-74.4000	168.3000	528	7.2	0.92	0.86	0.06	0.13	-25.11	-25.05	3.04	7.4	6.6	0.29	621
AP.003	-65.00925	-63.16279	623	7.4	1.20	1.14	0.06	0.18	-22.87	-24.43	3.58	7.4	11.5	0.63	629
AP.012	-63.48389	-60.2876	399	7.2	0.97	0.95	0.02	0.13	-24.10	-24.62	2.05	8.5	14.7	0.86	590
AP.035	-64.01938	-56.44577	223	7.1	0.63	0.52	0.11	0.07	-19.55	-23.43	2.51	8.7	11.5	2.10	372
AP.045	-63.44286	-57.2358	593	7.1	1.45	1.50	0.00	0.22	-20.49	-22.38	4.24	7.9	25.3	0.22	693
AP.046	-63.44296	-57.23574	706	6.9	1.60	1.63	0.00	0.23	-21.15	-22.26	3.73	8.3	14.1	1.00	623
AP.051	-63.41708	-56.49786	403	7.0	0.49	0.55	0.00	0.07	-20.98	-22.64	2.30	9.1	12.9	0.71	179
AP.057	-63.58397	-56.33443	395	7.0	0.77	0.70	0.07	0.10	-21.32	-22.75	2.59	8.2	15.0	0.58	425
AP.085	-62.53536	-59.08836	820	7.1	1.46	1.44	0.02	0.22	-22.89	-23.50	3.63	7.6	40.1	0.37	604
AP.106	-64.5158	-62.34183	497	7.1	0.56	0.60	0.00	0.08	-22.75	-23.59	2.33	8.7	26.7	0.75	583
AP.130	-64.38596	-62.14854	728	7.2	1.23	1.20	0.03	0.17	-23.97	-24.52	2.54	8.3	13.7	0.87	690
AP.136	-64.34361	-61.38131	390	7.3	1.13	1.07	0.06	0.15	-23.46	-23.77	3.45	8.3	11.8	0.63	260

Site Names	Al (mg/kg)	B (mg/kg)	Ba (mg/kg)	Cd (mg/kg)	Cr (mg/kg)	Cu (mg/kg)	Fe (mg/kg)	K (mg/kg)	Mg (mg/kg)	Mn (mg/kg)	Mo (mg/kg)	Na (mg/kg)	Ni (mg/kg)	P (mg/kg)	Pb (mg/kg)	Si (mg/kg)	Sr (mg/kg)	Ti (mg/kg)	V (mg/kg)	Zn (mg/kg)
WA.009	48.1	0.01	1.11	0.01	0.01	3.38	5.07	466	1124	0.21	0.00	9680	0.11	3557	0.07	91.2	0.58	0.84	0.16	0.15
WA.011	208	0.01	0.10	0.04	0.17	1.91	11.2	507	1109	0.26	0.01	9602	0.07	3170	0.02	136	0.89	0.27	0.24	0.04
WA.017	716	0.01	0.09	0.02	0.52	2.97	21.7	1025	1027	0.13	0.03	8955	0.06	2990	0.10	89.2	0.75	0.33	0.24	0.01
WA.021	238	0.01	1.21	0.02	0.05	3.56	12.4	975	491	0.71	0.00	4179	0.08	3555	0.03	85.9	0.27	0.37	0.18	0.08
WA.026	686	0.01	0.12	0.03	0.35	1.03	64.3	1148	1159	0.13	0.03	9731	0.06	3552	0.05	87.9	0.78	0.39	0.21	0.13
WA.031	482	0.01	0.43	0.04	0.16	2.21	55.6	985	1163	0.75	0.04	9736	0.10	3540	0.05	89.4	0.56	0.28	0.21	0.27
WA.057	52.4	0.01	0.04	0.02	0.02	2.28	6.14	764	689	0.43	0.02	5908	0.06	3107	0.04	68.6	0.39	0.59	0.20	0.02
WA.064	368	0.01	0.02	0.02	0.29	3.54	16.3	1003	1190	0.58	0.01	10590	0.07	3400	0.03	177	0.63	0.85	0.28	0.06
WA.068	416	0.01	0.08	0.03	0.08	3.93	17.0	965	391	0.57	0.03	3126	0.09	3634	0.02	58.6	0.41	0.46	0.24	0.15
WA.075	546	0.01	0.55	0.02	0.08	2.45	20.4	811	961	0.17	0.06	8031	0.08	3568	0.07	76.4	0.86	0.52	0.22	0.14
WA.098	179	0.01	0.03	0.01	0.07	1.91	7.82	328	430	0.72	0.00	3629	0.17	3701	0.09	68.2	0.87	0.41	0.12	0.23
WA.103	715	0.01	0.05	0.02	0.07	1.99	21.4	985	1006	0.25	0.00	8729	0.10	3601	0.04	87.5	0.62	0.73	0.15	0.12
WA.108	675	0.01	9.92	0.02	0.22	3.01	24.6	1075	1262	0.40	0.00	10660	0.11	3517	0.01	87.6	0.94	0.49	0.15	0.15
AP.003	93.7	0.01	0.47	0.34	0.44	5.56	11.3	577	1290	1.48	0.05	10720	0.08	3483	0.04	185	0.58	0.21	0.15	0.14
AP.012	554	0.01	0.25	0.03	0.23	0.66	49.6	1228	1183	1.01	0.14	9893	0.09	3477	0.07	209	0.95	0.32	0.17	0.07
AP.035	396	0.01	0.13	0.03	0.17	0.57	51.4	615	815	1.90	0.01	6848	0.11	3534	0.09	170	0.32	0.14	0.25	0.10
AP.045	729	0.01	0.13	0.03	0.41	0.66	63.7	1250	1351	1.74	0.00	11760	0.09	3364	0.07	211	0.21	0.31	0.24	0.07
AP.046	356	0.01	0.44	0.03	0.17	0.88	44.7	587	1236	0.75	0.00	10710	0.10	3434	0.04	181	0.28	0.42	0.22	0.13
AP.051	320	0.01	0.32	0.03	0.18	0.89	60.7	846	428	0.73	0.00	3797	0.12	3677	0.02	103	0.67	0.42	0.15	0.22
AP.057	1.03	0.01	0.06	0.02	0.04	0.50	0.28	4.2	895	1.16	0.03	7745	0.11	3549	0.04	172	0.45	0.32	0.25	0.10
AP.085	203	0.01	0.35	0.02	0.18	0.63	48.8	647	1242	2.15	0.00	10780	0.13	3461	0.12	183	0.64	0.63	0.25	0.21
AP.106	466	0.01	0.06	0.04	0.10	1.27	62.7	836	1130	1.88	0.09	9627	0.11	3367	0.05	266	0.82	0.33	0.09	0.06
AP.130	402	0.01	0.30	0.04	0.14	0.57	51.2	799	1352	0.49	0.03	11560	0.08	3360	0.03	241	0.51	0.51	0.28	0.03
AP.136	345	0.01	0.11	0.05	0.19	1.03	56.4	705	585	1.02	0.00	4766	0.12	3631	0.08	145	0.75	0.64	0.19	0.12

DL denotes parameter is below the detection limit

For isotope analysis, instrument standard deviation of 0.2 per mill for δ13C and 0.3 per mill for δ 15N. For the pH, %TOC and %N, accuracy is +/- 10% of the reported value. For NH_4-N, NO_3-N, S and elemental analysis, accuracy is +/- 5% of the reported value
