# Supplementary material for: Biogeochemical and Microbial Variation across 5500 km of Antarctic Surface Sediment Implicates Organic Matter as a Driver of Benthic Community Structure
Source: Front Microbiol. 2016 Mar 23;7:284. doi: 10.3389/fmicb.2016.00284 (PMC4803750; doi:10.3389/fmicb.2016.00284)
Supplement: Supplementary file 2 [file Table2.PDF]

Table S2. Correlations between taxonomic groups and geochemical variables

	**Taxonomic Identifier**	**Chemical parameter**	**Spearman Correlation***
Class	Crenarchaeota, Thaumarchaeota	pH	-0.611
		$\delta^{13}C_{org}$	-0.803
		NH_4^+-N	-0.667
		Si	-0.689
Phylum	Bacteroidetes	TOC	0.679
		$\delta^{13}C_{org}$	0.637
		Si	0.756
		NH_4^+-N	0.747
OTUs	Otu0000013, SAR	pH	0.599
		TOC	0.521
		$\delta^{13}C_{org}$	0.834
		NH_4^+-N	0.709
		Si	0.693
	Otu0000010, Stramenopiles	pH	0.697
		TOC	0.582
		$\delta^{13}C_{org}$	0.763
		NH_4^+-N	0.786
		Si	0.701
	Otu000001, Proteobacteria, Piscirickettsiaceae	$\delta^{13}C_{org}$	-0.582
		NH_4^+-N	-0.442**
		Si	-0.598
	Otu000002,Crenarchaeota, Cenarchaeaceae	$\delta^{13}C_{org}$	-0.622
		Si	-0.422**
	Otu000003,Crenarchaeota, Cenarchaeaceae	pH	-0.7
		TOC	-0.631
		$\delta^{13}C_{org}$	-0.776
		NH_4^+-N	-0.761
		Si	-0.712
	Otu000004,Proteobacteria, OM60	pH	0.626
		$\delta^{13}C_{org}$	0.562
		NH_4^+-N	0.611
		Si	0.606
	Otu000028,Bacteroidetes, Flavobacteriaceae	pH	-0.562
		TOC	-0.571
		$\delta^{13}C_{org}$	-0.634
		NH_4^+-N	-0.63
		Si	-0.533

Otu000059,Bacteroidetes, unknown	pH	0.524
	TOC	0.698
	$\delta^{13}C_{org}$	0.74
	NH_4^+-N	0.766
	Si	0.734
Otu000080,Bacteroidetes, Flavobacteriaceae	pH	0.547
	TOC	0.507**
	$\delta^{13}C_{org}$	0.824
	NH_4^+-N	0.624
	Si	0.698
Otu000097,Bacteroidetes, Flammeovirgaceae	pH	0.577
	TOC	0.563
	$\delta^{13}C_{org}$	0.637
	NH_4^+-N	0.683
	Si	0.606
Otu000145,Bacteroidetes, Flammeovirgaceae	TOC	0.652
	$\delta^{13}C_{org}$	0.737
	NH_4^+-N	0.83
	Si	0.682

*all significant at 0.01 level, two tailed, unless noted.

**significant at 0.05 level, two tailed
