# Supplementary figures and images for: Biogeochemical and Microbial Variation across 5500 km of Antarctic Surface Sediment Implicates Organic Matter as a Driver of Benthic Community Structure
Source: Front Microbiol. 2016 Mar 23;7:284. doi: 10.3389/fmicb.2016.00284 (PMC4803750; doi:10.3389/fmicb.2016.00284)

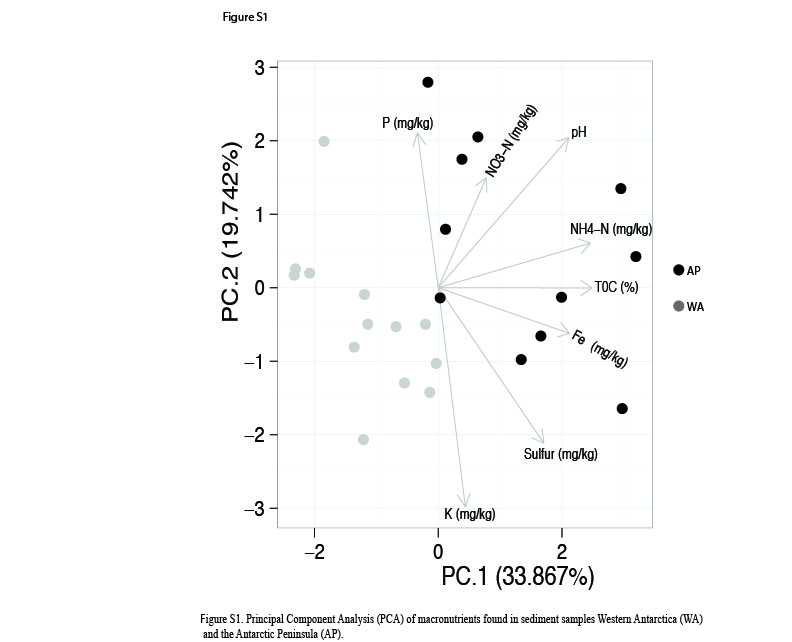

Supplement: Supplementary file 6 [file Image1.JPEG]

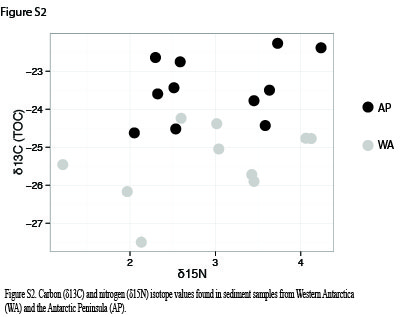

Supplement: Supplementary file 7 [file Image2.JPEG]

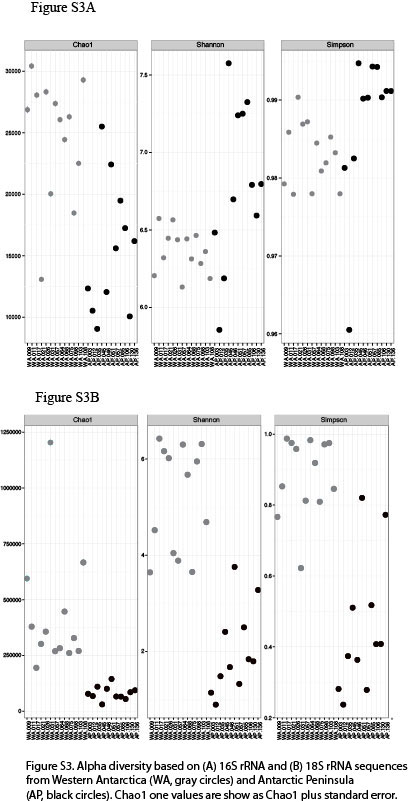

Supplement: Supplementary file 8 [file Image3.JPEG]

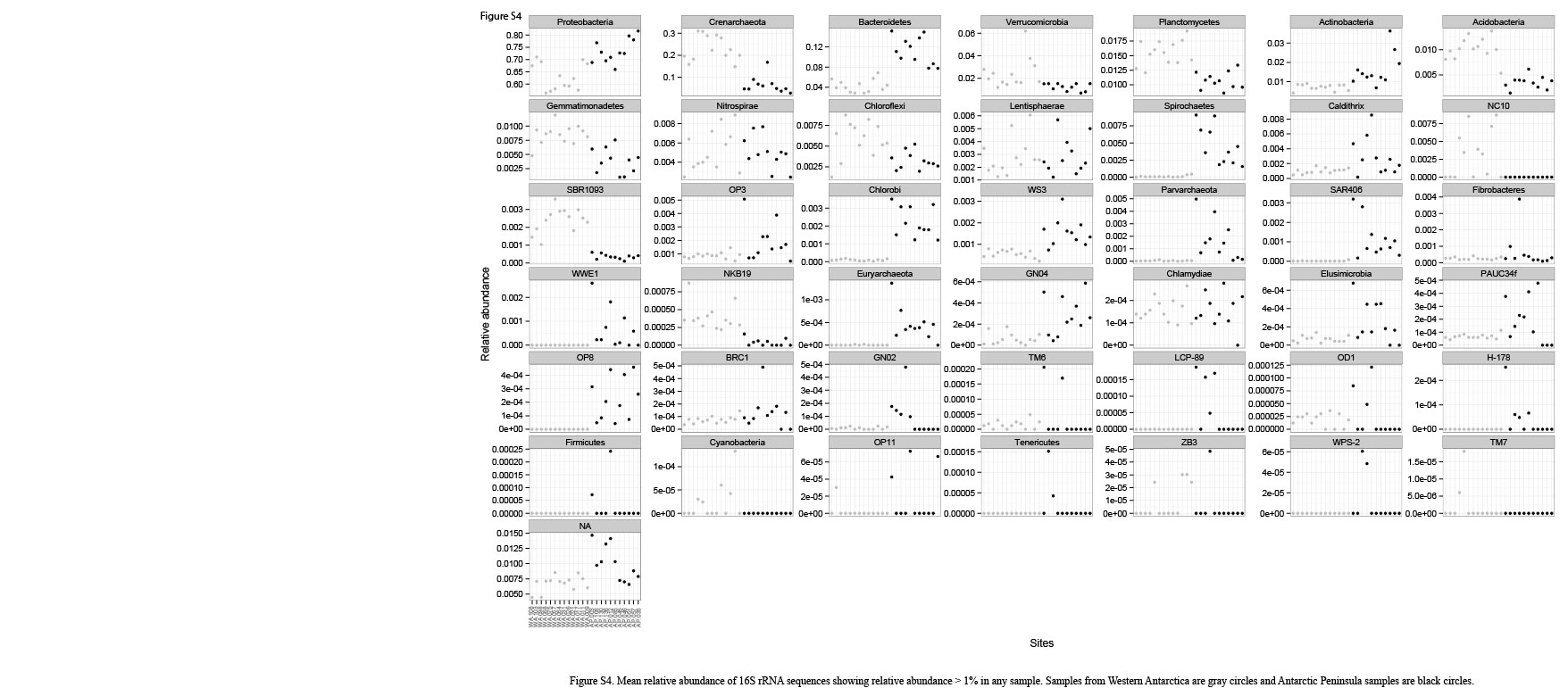

Supplement: Supplementary file 9 [file Image4.JPEG]
