# Supplementary material for: Biogeochemical and Microbial Variation across 5500 km of Antarctic Surface Sediment Implicates Organic Matter as a Driver of Benthic Community Structure
Source: Front Microbiol. 2016 Mar 23;7:284. doi: 10.3389/fmicb.2016.00284 (PMC4803750; doi:10.3389/fmicb.2016.00284)
Supplement: Supplementary file 10 [file Image5.pdf]

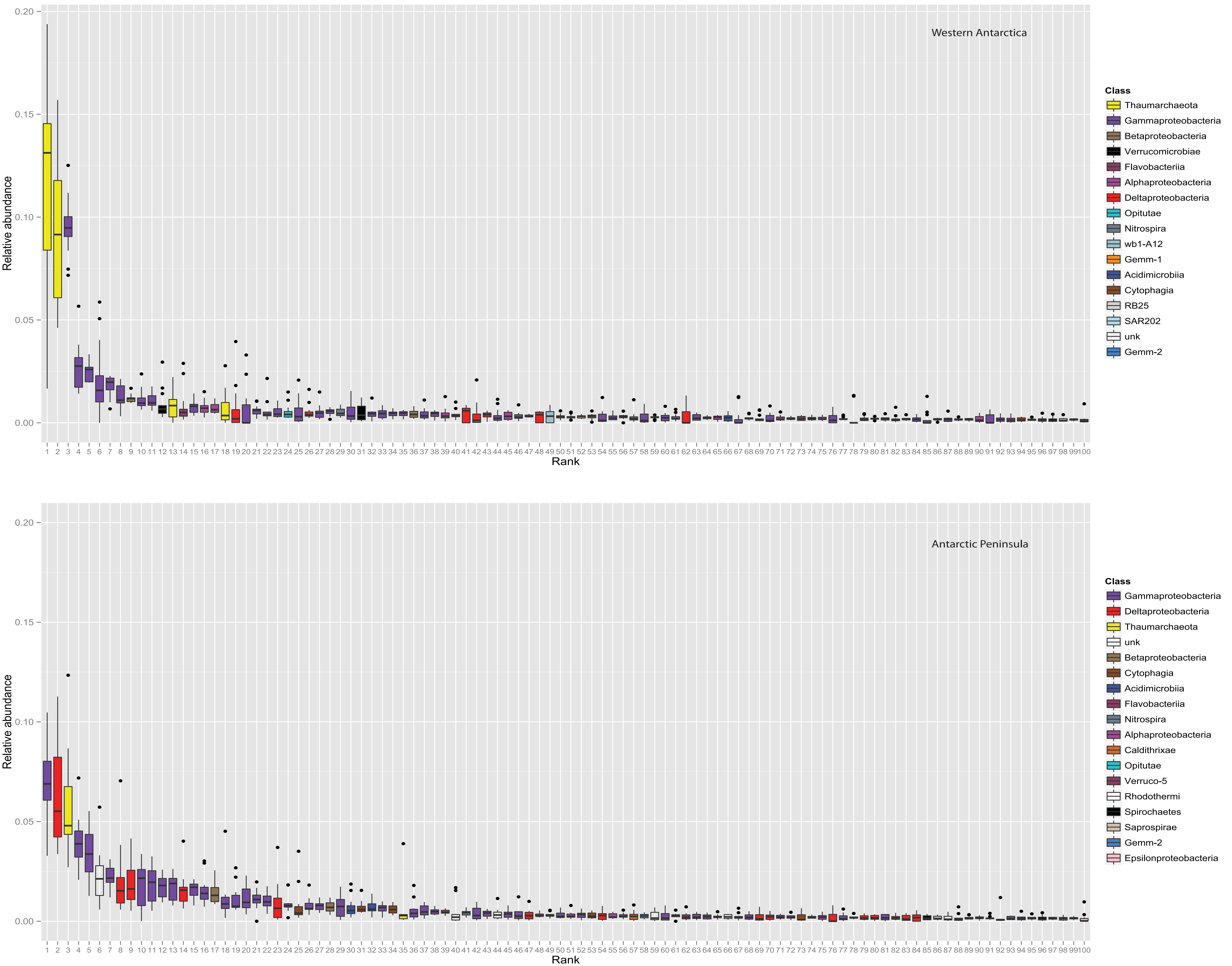

Figure S5A. Relative abundance box plot of the top 100 OTUs (based on mean) found in Western Antarctica (WA) or Antarctic Peninsula (AP) of 16S rRNA sequences. The dots represent outliers in the data.

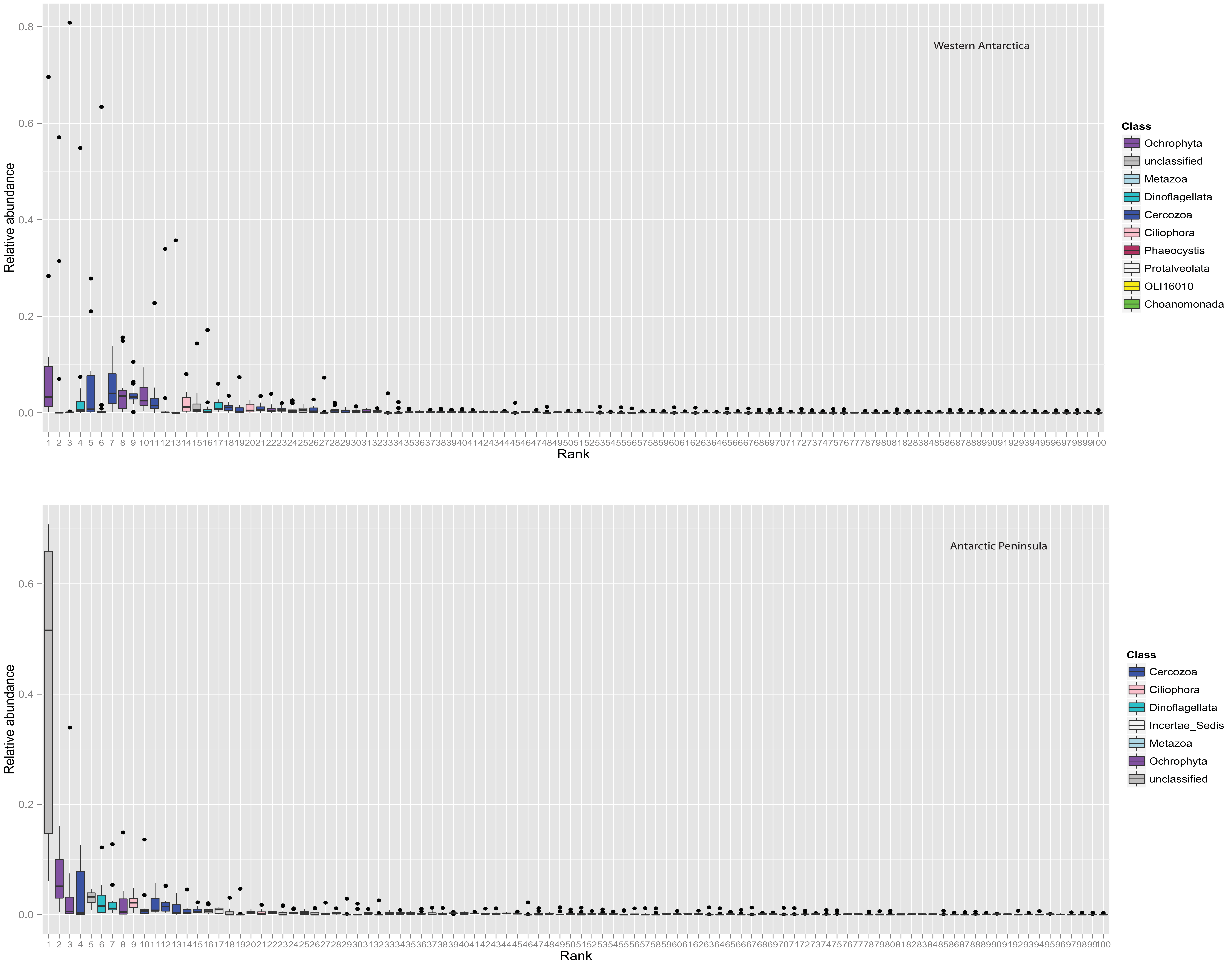

Figure S5B. Relative abundance box plot of the top 100 OTUs (based on mean) found in Western Antarctica (WA) or Antarctic Peninsula (AP) of 18S rRNA sequences. The dots represent outliers in the data.
